# Exploring Adipose Tissue Complexity Through Omics Approaches: Implications for Health and Disease

**DOI:** 10.3390/cells15050427

**Published:** 2026-02-28

**Authors:** Rajaa Sebaa

**Affiliations:** Department of Medical Laboratories, College of Applied Medical Sciences, Shaqra University, Shaqra 11961, Saudi Arabia; r.sebaa@su.edu.sa

**Keywords:** brown adipose tissue (BAT), white adipose tissue (WAT), beige adipose tissue (BeAT), omics approaches

## Abstract

**Highlights:**

**What are the main findings?**

- Omics revealed diverse adipocytes and their molecular structures and dynamics.
- Omics showed ATs coordinate whole-body metabolism via genes, proteins and metabolites.

**What are the implications of the main findings?**

- Omics could identify ATs-derived molecules as predictors of metabolic health.
- Omics may enable precision modulation of adipocyte subtypes to potentially treat metabolic disorders.

**Abstract:**

Adipose tissues (ATs) are dynamic and heterogeneous organs divided into three distinct categories, including white, beige, and brown ATs. Collectively, they contribute to systemic energy homeostasis in various ways. White adipocytes primarily store excess energy, whereas brown and beige adipocytes dissipate energy as heat through non-shivering thermogenesis. Recent advances in multi-omics technologies have transformed our understanding of adipocyte biology, enabling comprehensive interrogation of transcriptional, epigenetic, proteomic, and metabolomic networks that define adipocyte identity and function. Transcriptomic studies reveal distinct gene signatures underlying thermogenic activation and lineage commitment, while epigenomic profiling highlights regulatory elements that orchestrate adipocyte plasticity, particularly the inducible browning of white fat. Proteomic and metabolomic analyses further uncover mitochondrial remodeling, lipid turnover pathways, and metabolite, hormone interactions that regulate thermogenic capacity and metabolic health. Integrating these multi-layered datasets provides systems-level insights into the roles of environmental cues, such as diet and temperature, and endogenous factors, including hormonal signaling, circadian rhythms, and genetic background, in reshaping adipocyte phenotypes and influencing whole-body metabolism. Multi-omics approaches are increasingly identifying potential novel biomarkers and therapeutic targets aiming to enhance the activity of brown and beige adipocyte to combat obesity and metabolic disorders. Overall, these technologies provide a powerful framework for elucidating the complexity of ATs and advancing precision strategies for metabolic disease management and prevention.

## 1. Overview of Adipose Tissues

Adipose tissues (ATs) play vital roles in maintaining energy homeostasis, metabolic balance, and endocrine regulation. They are not merely passive energy-storage depots but rather dynamic, metabolically active, and thermogenic organs [1]. In addition to storing and releasing energy, ATs act as important endocrine regulators, communicating with other organs through the secretion of a wide range of bioactive molecules, collectively known as adipokines, including hormones, cytokines, and growth factors that influence inflammation, metabolism, appetite regulation, cardiovascular function, immune responses, and various other physiological processes [2]. Thus, ATs serve as a central hub in the coordination of systemic metabolic and hormonal networks, linking nutrient status to whole-body energy regulation and health.

**Figure 1:**
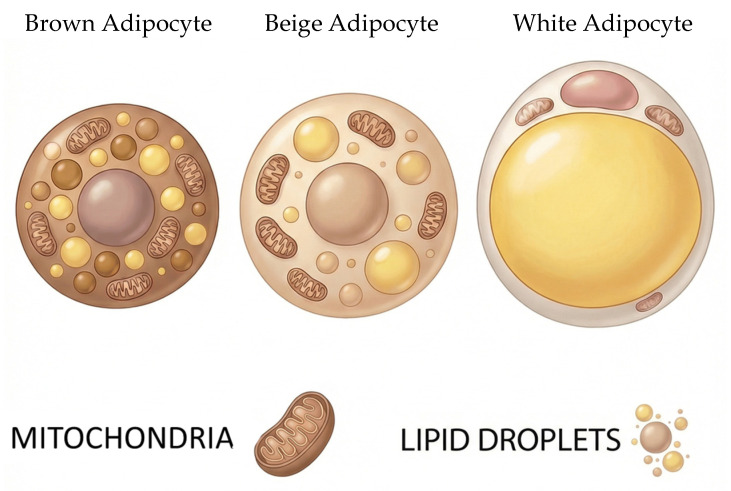
Morphological structure of Brown, Beige, and White Adipocytes.

Generally, there are three types of ATs including white adipose tissue (WAT), beige adipose tissue (BeAT), and brown adipose tissue (BAT). They are distinct with different morphological and functional characteristics, as well as unique metabolic properties and gene expression profiles, as briefly summarized in Table 1 and shown in Figure 1. WAT stores energy and regulates metabolism, BAT generates heat through thermogenesis, and BeAT acts as an inducible fat that can switch between energy storage and heat production [3,4,5]. They are distributed in various locations in the body as mentioned below. Each type of ATs exhibits different timing of appearance during development and posing distinct genetic origin [6]. Also, ATs are composed of distinct adipocyte populations with specialized structural and functional characteristics as demonstrated below. They are composed of various cell populations, including mature adipocytes, adipose-derived stem cells (ADSCs), preadipocytes and progenitor cells, adipose tissue macrophages (ATMs), endothelial cells, and other immune cells. The stromal vascular fraction (SVF), which includes ADSCs, ATMs, and endothelial cells, plays a crucial role in tissue remodeling, angiogenesis, and immune regulation. Furthermore, ADSCs contribute to the renewal and differentiation of new adipocytes, while ATMs maintain tissue homeostasis and modulate inflammation [7]. A wide range of traditional techniques has been extensively employed to investigate the structure, function, and contributions of these cells to physiological homeostasis and disease development. In addition, newly emerging techniques are providing novel insights into these processes, enabling deeper understanding of cellular mechanisms.

Particularly, this review aims to comprehensively summarize the current literature employing omics-based approaches including genomics, transcriptomics, proteomics, and metabolomics to elucidate the biology of ATs and their implications for health and disease. A literature search was conducted using PubMed, Google Scholar, and Web of Science databases to identify relevant studies published nearly in the last 10 years. The search was performed using keywords such as adipose tissues, adipocytes, epigenomics, genomics, transcriptomics, and metabolomics. Articles addressing background aspects of ATs and employing omics-based approaches were included to explore the complexity of ATs in human subjects as well as relevant animal and cellular experimental models. Studies were excluded if they did not directly examine ATs, lacked an omics-based analytical framework, focused on unrelated tissues or disease contexts, or provided insufficient methodological detail.

### 1.1. White Adipose Tissue (WAT)

#### 1.1.1. Anatomical Location of WAT

WAT is anatomically and functionally stratified into subcutaneous (sWAT) and visceral (vWAT) compartments, demarcated primarily by their distinct anatomical localizations [8]. sWAT depots are mainly located in the hypodermal layer beneath the skin, where they help maintain body temperature. In contrast, vWAT is found around vital organs within the thoracic and abdominal cavities, where it provides protection and serves as an accessible energy reserve to support the metabolic needs of these organs [1]. In homo sapiens, sWAT depots encompass regions including cranial, facial, abdominal, gluteal, and femoral ATs, whereas in murine models, analogous subcutaneous depots comprise the anterior subcutaneous and inguinal fat pads. Visceral adiposity across both taxa is characterized by discrete depots such as mesenteric, omental, retroperitoneal, and gonadal fat, each intimately associated with the visceral organs they envelop [9].

#### 1.1.2. Biological Function of WAT

WAT is a fat-storing tissue and represents the major type of AT found in the human body. It regulates energy balance, glucose and lipid metabolism, inflammation, and insulin sensitivity. WAT mainly consists of unilocular adipocytes containing a single large triglyceride droplet with relatively few mitochondria. Their triglyceride droplets are hydrolyzed to fatty acids during energy demands [3,10]. In addition, WAT also acts as an endocrine organ secreting adipokines, namely, leptin, adipsin, adiponectin, omentin, tumor necrosis factor-alpha (TNF-α), interleukin-6 (IL-6), monocyte chemoattractant protein-1 (MCP-1), plasminogen activator inhibitor-1 (PAI-1), resistin, visfatin, and retinol-binding protein 4 (RBP4) [11,12]. Briefly, adipokines regulate systemic metabolism by acting on key metabolic organs. For instance, leptin and adiponectin modulate hypothalamic STAT signaling to control appetite and energy expenditure and activate AMPK signaling in the liver and skeletal muscle, thereby suppressing hepatic glucose output, reducing inflammation, and enhancing glucose uptake and fatty acid oxidation [13,14]. In contrast, pro-inflammatory adipokines, including resistin, TNF-α, and IL-6, disrupt metabolic homeostasis by activating JNK and NF-κB pathways in the liver, leading to impaired insulin sensitivity and heightened systemic inflammation [15].

#### 1.1.3. Clinical Relevance of WAT

Evidence from clinical studies suggests that sWAT plays a protective role in maintaining metabolic health, yet its protective and metabolic function can be impaired under metabolic stress, including obesity, aging, sedentary lifestyle, or chronic low-grade inflammation, leading to fibrosis and immune-cell infiltration [16]. Briefly, in a state of stress, sWAT undergoes adipocyte hypertrophy, where individual fat cells enlarge beyond their optimal size, resulting in mechanical and metabolic strain. This hypertrophy triggers extracellular matrix (ECM) remodeling and deposition of fibrotic tissue, which stiffens the adipose structure and restricts further lipid storage. At the same time, dysfunctional sWAT attracts pro-inflammatory immune cells, including M1 macrophages and T cells, which amplify local inflammation, disrupting insulin signaling [17]. Furthermore, adipocytes from vWAT are more prone to lipolysis, releasing free fatty acids and inflammatory mediators directly into the portal circulation, which drains into the liver. This direct delivery enhances hepatic lipid accumulation, promotes insulin resistance, and stimulates pro-inflammatory signaling pathways that extend to peripheral tissues. Clinical and epidemiological evidence consistently shows that excessive vWAT is strongly correlated with type 2 diabetes, dyslipidemia, hypertension, cardiovascular disease, and certain cancers, highlighting its central role in metabolic risk. Furthermore, vWAT is more responsive to endocrine and paracrine signaling that promotes adipokine imbalance, oxidative stress, and systemic inflammation compared to sWAT [18,19].

### 1.2. Brown Adipose Tissue (BAT)

#### 1.2.1. Anatomical Location of BAT

In human infants, BAT is predominantly located in the interscapular, neck, axillary, and perirenal regions, with smaller depots found along the sternum and spine. These depots are highly active at birth, contributing to non-shivering thermogenesis and maintaining body temperature during the neonatal period. In adults, BAT is mainly localized to the cervical, axillary, and supraclavicular regions, with smaller deposits near the periaortic, perirenal, and paravertebral areas; although reduced in volume compared to infants, adult BAT remains metabolically significant and can be recruited in response to cold exposure or certain metabolic stimuli. In rodents, BAT depots are more variable, influenced by age, sex, and strain. The largest classical depot is in the interscapular region, with smaller depots located in the cervical, supraclavicular, and peri-aortic areas [20].

#### 1.2.2. Biological Function of BAT

BAT is a thermogenic form of ATs specialized in producing heat through a process known as non-shivering thermogenesis. BAT is composed of brown adipocytes that are rich in mitochondria and contain multiple small lipid droplets. Upon activation, BAT utilizes both glucose and fatty acids as primary energy substrates, oxidizing them within its mitochondria to drive thermogenesis. Brown adipocytes originate from the same progenitor of skeletal myoblast lineages, expressing myogenic factor 5-positive (*Myf5*^+^) precursors [21]. At the molecular level, the transcriptional co-regulator PR domain-containing 16 (PRDM16) acts as a master switch that directs these precursors toward brown adipocyte differentiation rather than myogenic cell fate under specific environmental and hormonal conditions. PRDM16 in brown adipocyte interacts with peroxisome proliferator-activated receptor gamma (PPAR-γ), which promotes the activation of brown adipocyte-specific gene expression patterns [22]. The thermogenic capacity of BAT is primarily mediated by uncoupling protein 1 (UCP1), which is embedded in the inner mitochondrial membrane. Mechanistically, UCP1 uncouples oxidative phosphorylation by allowing protons in the mitochondrial intermembrane space to return to the mitochondrial matrix without generating adenosine triphosphate (ATP), thereby dissipating the proton gradient as heat distributed throughout the body to maintain thermoregulation. The control of BAT activation is mainly under the sympathetic nervous system (SNS). Upon cold exposure or other stimuli, the hypothalamus triggers the release of norepinephrine (NE) from sympathetic nerve endings that innervate brown adipocytes. NE binds to β3-adrenergic receptors, leading to increased cyclic AMP (cAMP) levels and activation of protein kinase A (PKA). This cellular signaling cascade leads to the breakdown of lipid droplets, releasing free fatty acids that act as both energy substrates and activators of UCP1-mediated thermogenesis. Beyond its fundamental role in thermogenesis and thermoregulation, BAT exhibits significant effects on systemic energy metabolism by regulating the utilization and oxidation of glucose and fatty acids, which contributes to overall glucose and lipid homeostasis. Through promoting energy expenditure, BAT can improve insulin sensitivity, reduce circulating lipids, and mitigate excessive fat accumulations in the body. These metabolic effects of BAT point out its promising therapeutic potential for metabolic disorders, including obesity, type-2 diabetes, and other related conditions associated with impaired energy balance [3].

#### 1.2.3. Clinical Relevance of BAT

BAT holds substantial clinical importance in metabolic health, as its activation elevates energy expenditure, increases glucose utilization, enhances insulin sensitivity, and facilitates lipid clearance, thereby offering protective effects against obesity, type 2 diabetes, dyslipidemia, and metabolic syndrome [23]. Higher levels of BAT activity are typically correlated with lower body mass index and decreased visceral fat accumulation. Beyond its thermogenic function, BAT acts as an endocrine organ through the release of batokines such as FGF21, IL-6, and neuregulin 4, which modulate systemic metabolic processes, hepatic lipid metabolism, and inflammatory responses [24]. In addition, BAT plays an essential role in thermoregulation, especially in neonates, while diminished BAT function in older individuals may contribute to cold intolerance and reduced metabolic adaptability [25]. Owing to these metabolic and endocrine properties, BAT has become an attractive therapeutic target, with current approaches focusing on pharmacological stimulation via β3-adrenergic agonists, the use of thyroid hormone analogs and mitochondrial enhancers to boost oxidative metabolism, FGF21-based interventions to replicate its endocrine actions, cold exposure or cold-mimetic strategies to activate thermogenesis, and the promotion of WAT browning to expand overall thermogenic capacity. Taken together, BAT represents a key regulator of whole-body energy homeostasis and a promising avenue for innovative metabolic disease therapies.

### 1.3. Beige Adipose Tissue

#### 1.3.1. Anatomical Location of BeAT

Anatomically, BeAT is located in various areas in mice and humans. BeAT arises within WAT and is characterized by its metabolic flexibility and dynamic thermogenic capacity. Although BeAT shares the heat-producing function of BAT, it differs in its developmental origin. Unlike BAT, BeAT arises predominantly from myogenic factor 5-negative (*Myf5^−^*) precursors [21]. BeAT is generated primarily through the trans-differentiation of mature white adipocytes or the differentiation of specific *Myf5^−^* precursors cells within WAT depots into thermogenically active beige adipocytes [4,26]. 

#### 1.3.2. Biological Function of BeAT

BeAT consists of inducible thermogenic adipocytes that emerge within WAT depots. Unlike classical BAT, BeAT is not constitutively active but is recruited and activated under specific physiological conditions such as cold exposure, physical exercise, β-adrenergic stimulation, or hormonal signals [27,28]. Beige adipocytes have distinct gene expression patterns in comparison with brown or white adipocytes [29]. Its primary function is adaptive thermogenesis, generating heat to maintain body temperature. Beige adipocytes express uncoupling protein 1 (UCP1) in their mitochondria, which uncouples oxidative phosphorylation from ATP production, allowing energy derived from fatty acid and glucose oxidation to be dissipated as heat rather than stored as ATP. These cells are characterized by high mitochondrial content and metabolic flexibility, enabling them to switch from an energy-storing phenotype to an energy-dissipating state when activated. Additionally, beige adipocytes secrete signaling molecules that contribute to local and systemic metabolic communication. Beige adipocytes are intermittently present within sWAT depots, particularly in the inguinal and anterior subcutaneous regions in mice [30]. It is primarily localized to the supraclavicular and neck regions, with additional deposits detected along the spine [21].

#### 1.3.3. Clinical Relevance of BeAT

There is an increasing body of research on BeAT following its discovery in humans, as it differs from the classical BAT abundant in infants [31]. While infant BAT is constitutively active and crucial for thermogenesis, BeAT appears in adults within WAT depots in response to stimuli such as β3-adrenergic stimulation, cold exposure, and exercise, thereby serving as a potential target for enhancing energy expenditure and combating obesity in adults [32]. The inducible UCP1-expressing BeAT represents a promising target for combating obesity and metabolic disorders, such as atherosclerosis, arterial hypertension and diabetes mellitus type 2 [28].

## 2. The Traditional Approaches Used in the Study of ATs

In order to understand the physiology of ATs and their involvements in health and disease, a wide range of experimental tools have been developed and applied [33,34]. These tools enable the scientific exploration of ATs from multiple dimensions, including physiological, cellular, molecular, and metabolic levels. Furthermore, researchers have employed a variety of imaging, cellular, and molecular techniques [34,35,36,37,38,39]. Moreover, researchers have performed histological approaches, including light and electron microscopy, immunohistochemistry, and immunofluorescence, allowing visualization of adipocyte morphology, lipid content, and depot-specific markers [40,41,42]. In addition, non-invasive imaging modalities, such as positron emission tomography (PET), computed tomography (CT), and magnetic resonance imaging/spectroscopy (MRI/MRS), enable quantification of ATs volume, metabolic activity, and BAT thermogenesis in vivo settings [43,44,45,46,47,48]. At the cellular level, techniques such as primary adipocyte culture, flow cytometry, and functional assays assessing lipolysis, glucose uptake, and mitochondrial respiration offer valuable insights into the metabolic properties of various types of ATs [49,50,51]. Among these advanced methodologies, omics technologies have emerged as particularly powerful tools, offering a comprehensive and integrative understanding of ATs biology at the molecular level. Traditional methods such as electron microscopy, immunohistochemistry, immunofluorescence, PET, and flow cytometry provide valuable information on tissue structure, specific proteins, and localized cellular activity, but they are inherently limited to predefined targets and cannot capture the full complexity of ATs.

## 3. Omics Approaches Used in the Study of ATs

In contrast, omics approaches allow the simultaneous, system-wide analysis of thousands of genes, proteins, or metabolites, revealing regulatory networks, metabolic pathways, and signaling interactions that were previously hidden. Among these advanced methodologies, omics technologies have emerged as particularly powerful tools, offering a comprehensive and integrative understanding of ATs biology at the molecular level, as illustrated by examples of studies mentioned below. By providing an unbiased, holistic view, omics not only complements traditional techniques but also uncovers novel mechanisms, biomarkers, and therapeutic targets that single-target or imaging-based methods alone cannot detect [52].

Omics research emerged from rapid advancements in molecular biology, genomics, and high-throughput analytical technologies. Its foundation was established with the completion of the Human Genome Project (HGP) in the early 2000s, which provided the first complete map of the human genome and demonstrated the power of large-scale, system-wide molecular analysis. This milestone highlighted the limitations of studying individual genes or proteins in isolation and emphasized the need to investigate entire molecular networks to understand biological function and disease. Subsequent developments in high-throughput sequencing, mass spectrometry, and computational tools enabled Omics to expand into multiple layers, including epigenomics, transcriptomics, proteomics, lipidomics and metabolomics. Over the past two decades, omics has evolved into an integrative, multi-dimensional approach that links gene regulation, protein activity, metabolite dynamics, and cellular phenotypes, providing unprecedented insight into complex biological systems [53,54].

Building upon these advances, single-cell omics has emerged as a powerful extension of traditional bulk omics approaches by enabling molecular profiling at the resolution of individual cells. Unlike bulk analyses, which average signals across heterogeneous cell populations, single-cell omics captures cell-to-cell variability in gene expression, epigenetic regulation, protein abundance, and metabolic states. This approach has significantly advanced the understanding of cellular heterogeneity, rare cell populations, and dynamic biological processes in development, immunity, and disease, thereby providing a more precise and comprehensive view of complex biological system [55].

### 3.1. Epigenomics

Epigenomics studies heritable changes in gene function that do not involve alterations in the DNA sequence, including DNA methylation, histone modifications, and chromatin remodeling, all of which play critical roles in regulating tissue development, differentiation, and metabolic function [56]. Key techniques include bisulfite sequencing (BS-seq) for genome-wide DNA methylation analysis [57], chromatin immunoprecipitation followed by sequencing (ChIP-seq) for mapping histone modifications and transcription-factor binding sites [58], assay for transposase-accessible chromatin using sequencing (ATAC-seq) for profiling chromatin accessibility [59], and high-throughput chromosome conformation capture (Hi-C) for studying three-dimensional chromatin architecture and long-range genomic interactions [60]. These methods, particularly when applied at the single-cell level, can reveal heterogeneity among adipose cell populations and uncover regulatory networks that control gene expression in response to environmental, nutritional, and metabolic cues.

### 3.2. Transcriptomics

Transcriptomics focuses on analyzing the entire set of RNA transcripts expressed in a cell, tissue, or organism at a particular time, providing insight into the regulation of gene expression under physiological and pathological conditions [61]. Among the principal tools, RNA sequencing (RNA-seq) offers sensitive, high-throughput, genome-wide profiling of both coding and non-coding RNAs, while microarray analysis represents an earlier hybridization-based approach limited to predefined transcripts [62]. Single-cell RNA sequencing (scRNA-seq) enables transcriptome analysis at single-cell resolution, revealing cellular heterogeneity and distinct cell populations within complex biological systems [63]. Complementary techniques such as spatial transcriptomics preserve tissue architecture while mapping gene expression patterns, and quantitative real-time PCR (qRT-PCR) is commonly used to validate specific transcriptomic findings [64]. Together, these methods provide a comprehensive view of gene regulatory networks and molecular pathways that cannot be achieved using traditional targeted or imaging-based approaches alone.

### 3.3. Proteomics, Lipidomics, and Metabolomics

Proteomics, lipidomics, and metabolomics are complementary omics approaches that together provide a comprehensive and dynamic understanding of cellular function, regulation, and phenotype. Analytical platforms such as mass spectrometry (MS) and nuclear magnetic resonance (NMR) spectroscopy are central to all three approaches, enabling high-throughput, sensitive, and accurate profiling of proteins, lipids, and small-molecule metabolites. These analyses can be performed using untargeted approaches, which broadly survey all detectable molecules for discovery and hypothesis generation, or targeted strategies, which focus on the precise quantification of predefined molecules or pathways. Semi-targeted approaches, metabolic fingerprinting, and flux analysis further allow detailed exploration of functional dynamics, pathway activity, and molecular interactions across multiple omics layers.

Proteomics examines the complete set of proteins in a cell, tissue, or organism, including their abundance, interactions, and post-translational modifications, which are central to virtually all biological processes. Key tools include liquid chromatography–tandem mass spectrometry (LC–MS/MS), matrix-assisted laser desorption/ionization mass spectrometry (MALDI-MS), and two-dimensional gel electrophoresis (2D-GE), allowing high-resolution protein identification, quantification, and characterization of modifications such as phosphorylation and acetylation [65].

Lipidomics, a branch of metabolomics, analyzes the diverse array of lipid species involved in membrane structure, energy storage, and signaling. Techniques include LC–MS, GC–MS for volatile or derivatized lipids, MALDI-MS, and imaging MS, which enable detailed profiling of lipid composition, dynamics, and spatial distribution. Lipidomics uncovers subtle changes in lipid remodeling and signaling that are often invisible in protein- or gene-centered analyses [66].

Metabolomics focuses on small-molecule metabolites, reflecting the downstream products of gene and protein activity, and providing a direct link between genotype, environment, and phenotype. MS- and NMR-based methods allow simultaneous identification and quantification of hundreds to thousands of metabolites. Untargeted metabolomics captures broad metabolic patterns, while targeted and semi-targeted approaches focus on defined metabolites or classes, complemented by metabolic fingerprinting and flux analysis to study pathway dynamics [67].

When epigenomics, genomics, transcriptomics, proteomics, lipidomics, and metabolomics are integrated, they provide a multi-layered, system-level view of biological networks, connecting gene regulation, protein function, lipid composition, and metabolite dynamics. This holistic perspective allows researchers to uncover regulatory mechanisms, pathway crosstalk, and phenotypic outcomes that would remain hidden when using any single omics approach. By capturing information across multiple molecular layers, this combined strategy offers powerful insights into cellular function, disease mechanisms, and the effects of environmental, nutritional, or therapeutic interventions, enabling a more comprehensive understanding of complex biological systems.

## 4. Omics Studies of ATs

In the field of ATs, omics have been applied to identify key regulatory genes, signaling pathways, and metabolites involved in adipocyte differentiation, thermogenesis, metabolic adaptation, and intra-cellular communication under physiological or pathological conditions. Recently, single-cell and spatial omics approaches have allowed researchers to resolve cellular heterogeneity and map ATs-specific interactions at unprecedented level. This review provides an updated overview of the omics-related studies of ATs within the last ten years, with a focus on genomics, transcriptomics, proteomics, metabolomics, and spatial omics approaches using a wide range of experimental models in both physiological and pathological contexts.

### 4.1. Omics Studies of WAT

Omics approaches have been used to comprehensively understand the physiological and pathological roles of WAT. These approaches enable the identification of biomarkers, therapeutic targets, and mechanistic pathways linking WAT biology to systemic metabolic health. Importantly, omics studies in WAT have been conducted using a broad range of experimental models, including animal and human models. These studies clinically provide relevant insights into metabolic disease in relation to WAT. Examples of these studies are mentioned briefly here.

#### 4.1.1. Omics Studies of WAT in Animal and Cellular Models

Several animal studies have investigated WAT using omics approaches, both under basal conditions and following stimuli or pharmacological interventions, as summarized below. A mouse study has explored new insights into the biological heterogeneity of WAT. In this study, the authors aimed to characterize and compare the basal lipid composition and gene expression profiles of sWAT and vWAT in mice. They applied a high-coverage targeted lipidomics approach and identified 342 lipid species across 19 lipid classes, uncovering significant depot-specific differences; for instance, sWAT had relatively higher levels of low-abundance phospholipids, sphingolipids (like ceramides), and cardiolipins, whereas differences in triacylglycerol (TAG) and free fatty acid (FFA) composition between depots reflected variations in chain length and saturation. Network analysis showed that TAG and phospholipids formed separate subnetworks, hinting at distinct lipid interactions in each depot. Complementary transcriptomic analysis further revealed that genes differing between vWAT and sWAT were enriched for pathways related to lipid metabolism, insulin resistance, and inflammation. Taken together, their results emphasize that visceral and subcutaneous fat depots have fundamentally different lipid and transcriptional landscapes [68]. Another mouse study aimed to investigate the influence of sex and fat depot on the heterogeneity of progenitor cells in WAT. Researchers isolated progenitor cells from mouse visceral fat perigonadal and inguinal sWAT of both sexes and performed integrated proteomic, genetic and transcriptomic analyses. They found that progenitor subpopulations exhibit distinct molecular signatures depending on depot and sex, including differences in lipid metabolism, mitochondrial function, and inflammatory pathways. Key regulators such as Glutathione S-Transferase Mu 1 (GSTM1) and PPARγ phosphorylation were identified as contributing to depot- and sex-specific differences in adipogenic potential, providing a detailed atlas of progenitor-cell heterogeneity [69]. Additionally, omics approaches have been used to study the respond of WAT to diet, drugs, and other stimuli at the molecular and metabolic levels. A study investigated the effect of a high-fat-diet-induced insulin resistance and rosiglitazone treatment on WAT and liver in mice using integrated transcriptomics, proteomics, and metabolomics. They found that insulin resistance disrupted pathways such as the TCA cycle, oxidative phosphorylation, and amino acid metabolism, particularly in WAT. Rosiglitazone mainly restored PPAR signaling and mitochondrial function in WAT, while in the liver it altered vitamin B metabolism, with sphingosine-related metabolites marking treatment effects [70]. Furthermore, a mouse study aimed to identify proteins in WAT that distinguish obesity-prone (OP) from obesity-resistant (OR) mice under a high-fat diet and to determine the effect of a traditional herbal medicine (TH) on these proteins. By using 2D electrophoresis and MALDI-TOF mass spectrometry, the authors found 57 differentially expressed and identified protein spots between OP and OR mice, many of which were reversed by TH treatment. Key proteins included Annexin A1/5, FABP4, cofilin-1, and enzymes linked to metabolic regulation, suggesting these as potential markers of obesity susceptibility. TH treatment reduced weight gain and altered the expression of 45 of these proteins, supporting its anti-obesity effect via modulation of WAT proteome [71]. Moreover, a rat study aimed to investigate whether long-term combined exposure to bisphenol A (BPA) and fructose induces lipid remodeling in WAT. In their study, they used widely targeted quantitative lipidomics to profile the lipidome in rat WAT after six months of exposure, measuring 734 distinct lipid species. Multivariate analyses showed that rats exposed to high-dose BPA plus fructose had a clearly distinct lipid profile compared to controls and single-exposure groups. Key lipids driving the differences included specific phosphatidylcholines (PC) and various triacylglycerols (TGs). These findings suggest that chronic BPA and fructose co-exposure significantly remodels adipose lipid composition, pointing to potential metabolic disturbance in WAT [72]. Another mouse study explored the impact of obesity on reshaping the protein composition of exosomes, extracellular vesicles released by cells carrying proteins, lipids, and nucleic acids to mediate intercellular communication, released from different adipose depots. Using quantitative proteomics, the researchers found that WAT-derived exosomes were most strongly affected by obesity, with changes in proteins linked to lipid and energy metabolism. Functional experiments showed that these exosomes could alter systemic lipid profiles when introduced into recipient mice, highlighting a potential role for WAT-derived exosomes in mediating obesity-related metabolic changes [73]. Moreover, researchers from another animal study aimed to examine the impact of lipopolysaccharide (LPS)-induced acute inflammation on lipid metabolism and protein expression in WAT of growing pigs. They measured mRNA levels of genes related to inflammation and lipid metabolism, assessed lipolytic enzyme activity, and performed label-free quantitative proteomics. Their results showed that LPS triggered inflammatory signaling via TLR2/4 and increased pro-inflammatory cytokines. At the same time, expression of lipid synthesis genes (e.g., *ACACA*, *FASN*, *SCD*) decreased, lipolytic activity dropped, and 47 proteins changed in abundance (including decreased PRKAR2A and β-tubulin). Interestingly, the adiponectin and zinc-α2-glycoprotein were upregulated at the protein level. These findings suggest that systemic inflammation significantly suppresses lipid metabolism in WAT and alters its proteome in inflammation setting [74]. Another external factor, hawthorn ethanol extracts (HEE), were examined on WAT metabolomics. The authors aimed to examine HEE impacts on the metabolite profile of WAT in a rat model of hyperlipidemia. They used gas chromatography-mass spectrometry (GC-MS) for untargeted metabolomics on ATs samples, after confirming that HEE lowers blood lipids (cholesterol, triglycerides, LDL) in high fat diet-fed rats. Their analysis found a number of endogenous metabolites (like L-threonine, aspartic acid, glutamine, mannose, inositol, and oleic acid) that were disrupted by hyperlipidemia but partially restored by HEE. Pathway analysis highlighted changes in fatty acid biosynthesis, unsaturated fatty acid biosynthesis, glycerolipid metabolism, and amino acid metabolic pathways. These results suggest HEE helps correct lipid and amino acid metabolic disturbances in ATs under hyperlipidemic conditions [75]. In agreement within the same line, a genetic mouse model was used to investigate the effect of deletion of angiotensin-converting enzyme 2 (ACE2) on the global metabolism across multiple tissues, including WAT. Using untargeted metabolomics, they profiled serum, liver, skeletal muscle, and epididymal white adipose tissue (eWAT) in *ACE2* knockout (KO) and wild-type mice under normal diet and high-fat diet. They found hundreds of significantly altered metabolites in all compartments, including increases in N-phenylacetylglutamine, bile acids, eicosanoids, and steroid hormones, and reductions in several lysophospholipids and amino acid-derived metabolites. Pathway analysis showed tissue-specific dysregulation such as amino acid metabolism and glutathione metabolism in WAT. Together, the findings indicate that ACE2 deficiency triggers broad, tissue-dependent metabolic distress, and the knockout model was used to uncover the role of ACE2 in regulating metabolism and tissue sensitivity, especially in WAT [76].

#### 4.1.2. Omics Studies of WAT in Human Studies

Human studies have increasingly applied omics technologies to understand WAT biology. A study focused on human WAT samples from different depots was conducted to characterize lipid composition varies across different human adipose depots. Using an optimized lipidomics workflow on small subcutaneous fat biopsies collected from the abdomen, thigh, breast, and lower back of adults, the researchers performed high-resolution mass-spectrometry-based profiling and identified a broad range of lipid species. Their analysis showed clear depot-specific differences, with thigh ATs displaying a distinct enrichment of long polyunsaturated triglycerides compared with other sites. Overall, the work demonstrates that human ATs is not metabolically uniform and that regional lipid profiles may contribute to differences in metabolic function [77]. At the cellular level, a study was designed to map the full transcriptional diversity of human sWAT at single-cell resolution, including both adipocytes and non-adipocyte cell types. They used a full-length single-nuclei (sn) RNA-Seq approach on whole tissue and isolated adipocytes, along with single-cell RNA-Seq on the stromal vascular fraction (SVF). Their data revealed three distinct adipocyte clusters, traced adipocyte differentiation through pseudotime, and showed that SVF scRNA-Seq provides greater resolution of non-adipogenic cells. This work demonstrates a previously underappreciated level of cellular heterogeneity in human subcutaneous fat and underscores the value of full-length transcriptomics for deeper cell-type characterization [78]. Additionally, a single-cell-based study was performed to generate a detailed cellular map of human sWAT using full-length single-cell and single-nucleus transcriptomics. By profiling whole tissue, isolated adipocytes, and stromal vascular cells, the researchers identified multiple distinct cell populations, including several previously unrecognized adipocyte subtypes, along with diverse immune, endothelial, and progenitor cells. Their analyses also traced adipocyte differentiation pathways and showed that whole-tissue nuclei profiling captures far richer cellular heterogeneity than adipocyte-only sequencing. Overall, the work highlights the complex and heterogeneous nature of human sWAT [79]. Building on recent advances in transcriptomic technologies, by integrating single-nucleus RNA sequencing with bulk transcriptomics and clinical data, we constructed a detailed cellular atlas of sWAT and vWAT from individuals with metabolically healthy and unhealthy obesity. These approaches reveal that dysfunction of ATs, despite being a major determinant of metabolic risk, has been poorly understood at the level of specific cell populations and transcriptional programs. Our analyses identify mesothelial cells, adipocytes, and adipocyte progenitors as the cell types most strongly associated with metabolic disease, and uncover cell-specific programs, including mesothelial-to-mesenchymal transition, that may explain why obesity does not uniformly lead to metabolic dysfunction [80]. In addition, using single-cell transcriptomics and flow cytometry, we mapped the stromal vascular fraction of lean and obese human WAT, identifying new subsets and developmental trajectories of innate lymphoid cells, dendritic cells, and monocyte-derived macrophages that expand in obesity. Analysis of cell–cell interactions revealed a shift from immunoregulatory programs in lean WAT to pro-inflammatory networks in obese WAT, providing a detailed view of its homeostatic and inflammatory circuits [81].

Interestingly, Lange and colleagues aimed to build a detailed, high-confidence reference lipidome of human WAT to support studies of lipid remodeling in obesity. They applied tissue-tailored extraction and LC-MS/MS workflows to sWAT and vWAT samples from lean and obese individuals, identifying 1636 lipid molecular species qualitatively and semi-quantifying 737 species. Their deep lipidomic profiling revealed significant depot- and phenotype-specific changes, especially in sphingolipids, ether lipids, and neutral lipids, and unveiled a surprisingly diverse spectrum of ceramides. Their results were used to build a data-rich lipidome resource named AdipoAtlas that can serve as a scaffold for biomarker discovery, high-throughput lipidomic assays, and potentially clinical application [82]. Furthermore, these studies have shown the influence of disease, diet, and drug on WAT, revealing depot-specific differences and molecular changes in both health and disease. As in this study, researchers examined the protein cargo of WAT-derived extracellular vesicles (adiposomes) from visceral fat in 75 obese and 47 lean adults to uncover molecular signatures linked to cardiometabolic risk. Mass-spectrometry proteomics identified 64 differentially abundant proteins, including upregulation of inflammatory proteins (CRP, C9, APOC1) and downregulation of protective proteins (adiponectin, transthyretin, fibrinogen subunits) in obese individuals. Network analysis highlighted TNF and IL-1 as key upstream regulators, and machine-learning models using these protein signatures accurately classified obesity, diabetes, hypertension, dyslipidemia, and liver steatosis, indicating that adiposomes reflect systemic metabolic and vascular dysfunction in obesity [83]. Moreover, an untargeted metabolomic analysis comparing vWAT and sWAT revealed that the metabolic impact of obesity far outweighs differences attributable to fat depot location. The study showed that severely obese individuals exhibit a distinct amino acid metabolic profile, characterized by greater release of glutamine and alanine and reduced uptake of multiple essential and branched-chain amino acids, while depot-specific variation was minimal and limited to a single metabolite. These results indicate that obesity induces widespread metabolic reprogramming in ATs, suggesting that overall adiposity, rather than anatomical depot, is the primary determinant of adipose metabolomic patterns [84]. In the diabetic context, an exploratory study compared sWAT from obese diabetic and non-diabetic individuals undergoing bariatric surgery using an untargeted metabolomics approach. sWAT samples from 17 participants were analyzed with the untargeted metabolomics, identifying 421 metabolites across lipid, amino acid, and energy-related pathways. The analysis showed no statistically significant differences between diabetic and non-diabetic groups after multiple-comparison correction. Although a few metabolites showed small fold-changes, mainly in lipid and amino acid pathways, these differences were not robust. Overall, the study concludes that obesity itself likely masks diabetes-related metabolic alterations in sWAT, suggesting that this tissue may not be sensitive for distinguishing diabetes status in bariatric surgery patients and emphasizing the need for further targeted or larger-scale investigations [85].

Collectively, omics approaches have greatly advanced our understanding of WAT, revealing its remarkable heterogeneity, depot-specific metabolic and molecular signatures, and dynamic responses to diet, drugs, environmental exposures, and disease. Animal and human studies integrating lipidomics, transcriptomics, proteomics, and metabolomics have uncovered distinct adipocyte subtypes, progenitor cell diversity, extracellular vesicle-mediated signaling, and obesity- or inflammation-driven metabolic remodeling. Collectively, these findings highlight that overall adiposity, rather than anatomical depot, often dominates the metabolic landscape of WAT, and that obesity can mask more subtle disease-specific alterations, such as those associated with diabetes. Such insights emphasize the importance of multi-omics approaches for identifying biomarkers, therapeutic targets, and mechanistic pathways, while also underscoring the need for larger, targeted, and high-resolution studies to fully capture WAT’s complexity in health and disease.

### 4.2. Omics Studies of BAT

Omics technologies provide a systems-level view of molecular changes in brown adipocytes, integrating transcriptomics, proteomics, and metabolomics to capture the response of these cells to thermogenic stimuli. By mapping gene expression, protein abundance, and metabolite dynamics simultaneously, multi-omics studies reveal the coordinated regulation of lipolysis, mitochondrial activity, and energy metabolism, offering insights into the mechanisms controlling heat production and potential targets for metabolic interventions. These studies often utilize a variety of models, including cultured brown adipocytes, primary cells, and in vivo animal models, to capture both cellular and systemic aspects of thermogenic regulation. Several studies addressing this focus are summarized below.

#### 4.2.1. Omics Studies of BAT in Animal and Cellular Models

Recently, a study conducted on thermogenic lipolysis in brown adipocytes used a multi-omics approach integrating transcriptomics, proteomics, and metabolomics to map dynamic molecular changes after norepinephrine stimulation. At 4 h, cells exhibited acute metabolic stress with rapid mobilization of lipid stores, increased translation machinery, shifts in central carbon metabolism, and transient peaks in fatty acids like oleic and arachidonic acid, while some TCA intermediates and amino acids decreased. By 24 h, the cells transitioned to a sustained lipolytic state with enhanced β-oxidation, upregulation of protein synthesis, and adaptive reprogramming of energy metabolism; transcripts for TCA and electron transport were downregulated, while ribosomal genes increased. Integrated analyses identified arachidonic acid and protamine as potential regulators of lipolysis, highlighting coordinated, system-wide responses across molecular layers. Overall, the study demonstrates that thermogenic lipolysis is a tightly orchestrated, multi-layered process, with early acute stress adaptations followed by sustained metabolic reprogramming, providing insights into potential targets for modulating brown fat thermogenesis [86]. In this study, the authors subjected mice to a two-week cold-exposure (CE) protocol (4 °C, 4 h/day) and then used untargeted metabolomics via gas chromatography–mass spectrometry (GC-MS) to profile metabolic changes across multiple tissues, including BAT, WAT, serum, liver, spleen, and kidney. They detected dozens of significantly altered metabolites in each tissue (32 in BAT, 17 in WAT, 21 in serum, etc.), with many changes in amino acids, fatty acids, and nucleotides. Pathway-level analysis identified 12 metabolic pathways significantly affected by cold exposure, notably those related to amino-acid metabolism, fatty-acid metabolism, and energy metabolism. Their results suggest that cold exposure triggers broad, coordinated metabolic remodeling not only in BAT but across multiple organs, offering a systemic view of cold adaption reshaping whole-body metabolism [87]. A generated comprehensive and time-resolved molecular atlas of mouse BAT was conducted by integrating transcriptomic (RNA-seq) and metabolomic profiling from embryonic stages through to adulthood. They discovered two distinct developmental phases that are specific to BAT, rather than shared with other organs, highlighting that brown fat has a unique maturation program. Through their analysis of transcription factor binding sites, they further identified key regulatory transcription factors that likely drive these stage-specific programs. By comparing their data to other organ-development omics datasets, they demonstrated that both transcriptomic and metabolomic profiles of BAT are markedly different from those of other tissues, underscoring the specialized functional maturation of brown fat. Their findings provide not only a foundational “map” of the development of brown adipocytes and their metabolic changes over time, but also suggest important transcriptional regulators and metabolic pathways that could be targeted to modulate BAT function in metabolic disease [88]. A study was conducted on mouse interscapular BAT analyzing BAT-related proteins using 2D liquid chromatography–mass spectrometry, identifying 4949 proteins, with 4495 quantified by iBAQ. Proteins were classified by abundance: high-abundance proteins were enriched for mitochondrial oxidative phosphorylation and fatty-acid oxidation, middle-abundance proteins for protein synthesis, and low-abundance proteins for regulatory and apoptotic functions. Nearly 500 proteins were predicted to be secreted, highlighting a potential BAT secretome. This work provides a comprehensive proteomic map of BAT, serving as a reference for studies on thermogenesis and metabolic regulation [89]. A study was performed to use proteomics across six metabolic tissues and plasma to investigate systemic metabolic adaptations to cold exposure, quantifying over 11,000 proteins. In BAT, cold triggered selective remodeling of glycolysis and pentose-phosphate pathway, enhancing oxygen consumption and likely increasing UCP1 activity via reactive oxygen species. Concurrently, plasma proteome changes suggested coordinated cross-tissue communication, with cold-induced lipolysis in WAT and glucose production in the liver supporting BAT fuel requirements. These findings highlight that cold adaptation involves integrated, multi-tissue metabolic rewiring, linking BAT glucose utilization to systemic energy homeostasis and revealing mechanistic parallels with metabolic states such as obesity [90]. A further study was performed on newborn goats that were exposed to cold (6 °C) for 24 h to investigate BAT responses. Perirenal BAT was analyzed using histology, Western blot, RNA-seq, and lipidomics. Cold exposure induced thermogenic activation, evidenced by increased UCP1 and PGC1α protein levels and histological changes consistent with lipid mobilization. Transcriptomic analysis revealed 1689 differentially expressed genes, including upregulation of thermogenesis-related genes, mitochondrial TCA cycle enzymes, and fatty-acid elongation and transport genes. Lipidomic profiling detected 1469 lipid species, with significant changes in triglycerides, diglycerides, and phospholipids, including increased phosphatidylcholine and phosphatidylethanolamine. The coordinated gene and lipid changes indicate that cold exposure triggers lipid remodeling, enhances mitochondrial function, and activates thermogenesis in goat BAT, highlighting complex metabolic and structural adaptations in response to cold [91]. Researchers used mass spectrometry-based proteomics to characterize lipid droplets (LDs) isolated from BAT of cold-exposed mice. Their analysis revealed that cold induced substantial remodeling of the LD proteome, including enrichment of lipolytic enzymes such as ATGL and HSL and increased levels of structural proteins PLIN1 and PLIN2. Remarkably, LDs from cold-treated BAT also contained mitochondrial proteins, including UCP1, suggesting close physical and functional associations between LDs and mitochondria. These findings indicate that cold exposure promotes LD remodeling and LD–mitochondria coupling, facilitating efficient fatty acid transfer to mitochondria to support β-oxidation and thermogenesis [92]. Researchers demonstrated that ceramides are critical regulators of thermogenic adipocyte function using mass spectrometry-based lipidomics to profile BAT lipids in genetically modified mice. Depletion of ceramides via deletion of the ceramide-synthesis gene *Sptlc2* enhanced mitochondrial respiration, increased energy expenditure, improved glucose homeostasis, and conferred resistance to diet-induced obesity. Conversely, ceramide accumulation through deletion of the ceramide-degradation gene *Asah1* impaired BAT thermogenesis, reduced mitochondrial efficiency, and decreased systemic energy expenditure. Mechanistically, ceramides suppressed lipolysis and glucose uptake in brown adipocytes, highlighting their direct role in limiting thermogenic and metabolic activity. These findings establish that ceramides are both necessary and sufficient to modulate BAT function and energy homeostasis [93]. A mouse study was performed to analyze BAT from mice fed a high-fat diet (HFD) for four weeks by RNA sequencing to assess transcriptomic changes induced by diet-induced obesity. HFD-fed mice exhibited increased body and BAT mass, and RNA-seq identified 357 differentially expressed genes, with 92 upregulated and 265 downregulated. Upregulated genes were enriched in immune and inflammatory pathways, including leukocyte chemotaxis and T-cell-mediated responses, while downregulated genes were associated with muscle structure, ion transport, and calcium signaling. Fatty-acid–binding proteins were elevated, whereas mitochondrial components such as Cox6a2 were decreased, suggesting altered lipid handling and potential mitochondrial impairment. Classic thermogenic genes (e.g., Ucp1, Ppargc1a) showed no significant change, indicating that short-term obesity triggers inflammatory and metabolic remodeling in BAT without major suppression of basal thermogenic gene expression [94]. An untargeted metabolomic study was performed to analyze WAT and BAT and to determine the impact of circadian disruption that alters metabolic homeostasis in mice. Their findings showed broad changes in lipid, amino-acid, and energy-related metabolites, with white fat displaying disrupted lipid handling and brown fat showing alterations linked to mitochondrial and thermogenic pathways. Notably, many metabolites that normally fluctuate across the day lost their rhythmicity, demonstrating that an intact circadian system is essential for maintaining the metabolic organization of ATs [95]. A study combined RNA-seq transcriptomics and mass-spectrometry-based lipidomics to comprehensively characterize BAT remodels and its lipid metabolism during cold exposure. Their integrative approach revealed extensive reprogramming of glycerolipid pathways, including coordinated changes in gene expression and lipid species involved in triglyceride synthesis, lipolysis, and fatty acid remodeling. Cold-induced changes were seen in glycerolipid metabolites within BAT, including shifts in key triacylglycerol species such as TAG (52:3) and TAG (54:4), elevations in diacylglycerols and monoacylglycerols linked to active lipolysis, and remodeling of phospholipids like PC (34:1) and PE (38:4). They also reported changes in lysophospholipids and several thermogenic fatty acids, highlighting a coordinated reorganization of lipid pathways that supports cold-driven metabolic activation [96]. A research group applied mass spectrometry-based lipidomics to characterize in detail the lipid composition of BAT in male versus female mice. Their analyses revealed a pronounced sex-specific lipid signature: male and female BAT differed substantially in the relative abundance of various lipid classes, including differences in fatty-acid composition and glycerophospholipids. For example, levels of certain fatty acids (such as stearic and arachidonic acid) and glycerophospholipid species varied depending on sex. These findings indicate that BAT lipid metabolism and composition are not identical between sexes, suggesting that male and female BAT may differ in membrane properties, fuel usage, or thermogenic capacity. The study underscores the importance of considering sex as a biological variable when investigating ATs lipidomics and metabolic function [97].

#### 4.2.2. Omics Studies of BAT in Human Studies

In this study, researchers applied mass-spectrometry-based proteomics to map and compare the secreted protein profiles (“secretomes”) of cultured human brown adipocytes (BAT-derived) versus white adipocytes. Using a multiplexed selective-ion monitoring (pmSIM) workflow, they quantitatively profiled proteins released into the culture medium as well as those retained in the cells. Their analysis revealed a distinct set of secreted proteins that are enriched in brown adipocytes, but not in white adipocytes, among them they identified EPDR1 as a novel “batokine.” Functional validation suggested that EPDR1 is secreted by brown adipocytes and may play a role in BAT-specific physiology. Altogether, the study demonstrates that human brown and white adipocytes have markedly different secretory signatures, suggesting that BAT may exert endocrine or paracrine effects through BAT-derived proteins rather than solely through classical metabolic outputs [98]. A human study was conducted using mass-spectrometry-based proteomics to compare human BAT with SAT, quantifying about 2500 proteins. They identified 318 proteins significantly enriched in BAT, most of which were mitochondrial, including UCP1 for uncoupled respiration, mitochondrial creatine kinases CKMT1A, CKMT1B, and CKMT2 for coupled ATP-synthase-linked respiration, and components of the mitochondrial interactosome such as the ADP/ATP translocator, phosphate carrier, and ATP synthase complex. Functional assays in human brown adipocytes confirmed that both coupled and uncoupled respiration contribute substantially to oxygen consumption, demonstrating that human BAT utilizes parallel energy-expenditure pathways to support human BAT thermogenesis [99]. Another human study was performed with the recruitment of participants exposed to mild cold to activate BAT, and mass-spectrometry-based metabolomics was used to profile serum metabolites before and after exposure. The analysis revealed widespread changes in circulating metabolites, with notable alterations in NAD^+^ metabolism, including increased levels of nicotinamide, tryptophan-derived intermediates, and other related cofactors. In addition to classical lipid and fatty-acid metabolites, these findings suggest that BAT activation influences systemic energy metabolism through modulation of NAD^+^-dependent pathways. The results highlight a potential link between BAT thermogenesis and broader metabolic regulation in humans, suggesting that BAT may contribute to whole-body metabolic homeostasis not only through heat production and lipid utilization but also by modulating key redox and cofactor networks [100]. Altogether, these studies illustrate that BAT function and thermogenesis are orchestrated through complex, multi-layered molecular networks that span transcripts, proteins, metabolites, and lipids. Multi-omics approaches reveal tightly coordinated regulation of lipid mobilization, mitochondrial activity, and energy metabolism in response to cold or adrenergic stimulation, highlighting both acute stress adaptations and longer-term metabolic reprogramming. Proteomic and lipidomic analyses demonstrate dynamic remodeling of mitochondrial proteins, lipid droplets, and secreted factors, while metabolomics uncovers tissue-specific and systemic shifts in amino acids, fatty acids, and NAD^+^-related cofactors. Integrating data across cellular, tissue, and organismal models underscores the interconnection between BAT and systemic metabolism, identifies potential regulators of thermogenesis, and provides a foundational resource for targeting BAT to improve energy balance and treat metabolic diseases.

### 4.3. Omics Studies of BeAT

Beige adipocytes, thermogenically active cells within WAT, play a key role in energy balance. Omics approaches have enabled system-level analyses of beige fat, revealing genes, proteins, and metabolites that regulate its differentiation, activation, and thermogenic function. These approaches provide integrated insights into the molecular mechanisms of beige fat and highlight potential targets for obesity and metabolic disease interventions. Regulation of beige adipocyte formation and thermogenic activity is critical for energy homeostasis and protection against metabolic disease.

#### 4.3.1. Omics Studies of BeAT in Animal and Cellular Models

In this study, researchers investigated the influence of PPARγ and the long noncoding RNA (lncRNA) Lexis on the balance between white and thermogenic beige adipocytes under thermoneutral conditions and during diet-induced obesity. Using genetic deletion and pharmacological inhibition of Lexis in mice, they demonstrated that loss of this lncRNA enhances both UCP1-dependent and UCP1-independent thermogenesis in white fat, increases energy expenditure, protects against obesity, and improves insulin sensitivity. Single-nucleus transcriptomic analyses identified a Lexis-regulated subpopulation of thermogenic adipocytes, and mechanistic studies revealed that Lexis modulates thermogenic programs through its interaction with TCF7L2, a transcription factor in the WNT signaling pathway [101]. A study was carried out transcriptomics (RNA-seq) of various vWAT depots in healthy mice to compare their gene expression profiles. Their analysis revealed that perinephric ATs stands out among visceral fat depots by exhibiting selectively high expression of beige-adipocyte marker genes, including thermogenic and mitochondrial genes such as *Ucp1*, *Cox7a1*, *Cox8b*, and *Cpt1b*, all characteristic of brown or beige adipocyte function. In contrast, expression of classical white-fat marker genes remained similar across depots, indicating that perinephric fat retains a white-fat identity while also harboring beige-like features. Quantitative PCR (qPCR) validation confirmed the RNA-seq findings, reinforcing that perinephric fat uniquely expresses a beige fat-like gene program under basal conditions. These results suggest that perinephric ATs may represent a previously underappreciated visceral fat depot with intrinsic beige adipocyte potential, which could have distinct physiological and pathological implications [102]. A research group investigated the impact of aging on beige adipocyte biogenesis and metabolic reprogramming using cold exposure, lineage-tracing, and single-nucleus transcriptomics (snRNA-seq) in murine inguinal WAT. Cold exposure elicited robust induction of multilocular, UCP1-positive beige adipocytes in young mice, whereas aged mice exhibited markedly attenuated beige adipogenesis and thermogenic gene expression. Lineage-tracing analyses demonstrated a diminished contribution of PDGFRα-containing adipocyte progenitors to newly formed beige adipocytes in aged animals, despite comparable in vitro differentiation potential. Single-nucleus transcriptomic profiling revealed age-dependent blunting of cold-responsive adipocyte subpopulations and upregulation of Npr3, a negative regulator of browning. Collectively, these findings indicate that aging impairs cold-induced beige adipocyte formation and associated metabolic remodeling, implicating progenitor dysfunction and altered transcriptional programs as central mechanisms [103]. An investigation was conducted to examine the molecular mechanisms underlying WAT browning (BeAT) by applying time-resolved proteomics to mice undergoing cold-induced thermogenic stimulation. Using mass spectrometry-based quantitative proteomic profiling, they analyzed dynamic changes in protein abundance in sWAT as it transitioned toward a beige phenotype. Their study identified nearly 500 proteins whose expression was significantly altered during browning, including metabolic enzymes, mitochondrial proteins, and regulators of lipid metabolism. Integrative analyses revealed that the transcription factor YBX1 is essential for driving thermogenic gene expression and promoting beige adipocyte commitment. Functional experiments confirmed that YBX1 loss impaired induction of UCP1 and other thermogenic markers, demonstrating that WAT browning involves coordinated proteomic remodeling, with YBX1 serving as a key regulator linking protein-level changes to functional thermogenesis [104]. A recent study investigated whether Gamma-Aminobutyric Acid (GABA) promotes beige adipocyte formation in high-fat-diet-induced obese mice by modulating gut microbiota and systemic metabolism. Using serum metabolomics, histology, immunostaining, gene expression analysis (*Ucp1*, *Pgc1α*, *Prdm16*, *Cidea*), and 16S rRNA sequencing, the researchers found that GABA reduced adiposity and inflammation, enhanced thermogenic/beige markers in iWAT, and induced beige adipocyte morphology. GABA also reshaped gut microbiota, increasing beneficial taxa (e.g., Akkermansia) and altering circulating metabolites, including elevated ketone bodies. Fecal microbiota transplantation confirmed that microbiota changes were necessary for beige adipocyte induction, highlighting a gut microbiota–metabolome–beige fat axis and suggesting a potential therapeutic strategy for obesity [105].

#### 4.3.2. Omics Studies of BeAT in Human Studies

A research study investigated the effects of metabolic bariatric surgery on the protein landscape of abdominal sWAT, containing beige adipocytes, in people with severe obesity. The authors analyzed biopsies from 46 patients before and six months after surgery and compared them with 15 healthy control subjects using high-resolution proteomics. They found that bariatric surgery leads to significant changes in the abundance of a subset of proteins, specifically, 46 proteins were upregulated and 34 were downregulated post-surgery. Before surgery, individuals with severe obesity showed decreased levels of proteins linked to mitochondrial integrity, amino acid breakdown, and lipid metabolism relative to controls; after surgery, there was increased representation of pathways involved in mitochondrial function, protein synthesis and folding, cytoskeleton regulation, and DNA binding/repair. These proteomic shifts suggest that weight loss after bariatric surgery is accompanied by broad changes in cellular metabolic and structural pathways, potentially underlying the metabolic improvements seen clinically and offering targets for future therapies for obesity and its complications [106].

Researchers conducted a randomized cross-over study in humans to investigate the impact of acute cold exposure on the circulating triacylglycerol (TAG) species. Using mass spectrometry–based lipidomics, they profiled plasma TAGs in detail and assessed the role of intracellular lipolysis through pharmacological inhibition. Cold exposure induced rapid and dynamic changes in specific TAG species, particularly those containing long-chain fatty acids, supporting thermogenic activity. These changes were abolished when intracellular lipolysis was blocked, demonstrating that the cold-driven remodeling of circulating TAGs depends on active lipid mobilization. The study highlights the critical role of intracellular lipolysis in shaping the human plasma lipidome during cold exposure [107]. Interestingly, a computational study developed a framework to quantify the browning potential of human WAT using deep-learning approaches applied to transcriptomic datasets. The researchers analyzed RNA-seq and microarray profiles from various human adipose samples to identify gene expression patterns characteristic of beige or brown adipocytes. Using an autoencoder-based model and ensemble machine-learning methods, they created a human browning capacity index (HBI) that integrates the expression of key browning-associated genes, including *DHRS11*, *REEP6*, and *STX11*. Application of this index to clinical adipose samples revealed that higher HBI scores corresponded with enhanced thermogenic and lipid-metabolic gene expression and better insulin sensitivity. This work provides a scalable, data-driven approach to assess the thermogenic potential of human fat, enabling quantitative evaluation of browning in both research and clinical contexts [108].

Collectively, omics studies have provided a broad, system-level understanding of the genes, proteins, and metabolites that regulate beige fat formation and function. These studies reveal molecular networks that could control thermogenesis, adipocyte differentiation, and metabolic activity, offering insights into potential strategies to improve energy balance and combat metabolic diseases.

## 5. Limitations and Future Directions

Despite the benefits gained through advances in omics technology, there are continuing weaknesses associated with the application of these technologies to provide an understanding of ATs. As a result of the fact that most studies have been carried out in mouse models under conditions that are not necessarily representative of what occurs in humans; therefore, many studies do not translate well into human physiology. The complexity and dissimilarity of the types of ATs, along with the diversity of cells present in each adipose depot, will limit the application of traditional bulk transcriptomic and proteomic methodologies; therefore, there remains a need to develop and use single cell and spatial omics analysis methodologies to assist with disentangling these challenging variables. Additionally, omics-based studies of ATs are associated with distinct bioinformatics challenges due to its cellular heterogeneity, high lipid content, and broad dynamic range of molecular abundances. In transcriptomics, normalization is complicated by large differences in RNA content across cell types and by sparsity in single-cell data. Lipidomics analyses are hindered by dominant high-abundance lipid species, missing values for low-abundance lipids, and strong batch effects, while proteomics faces limited coverage of low-abundance signaling proteins in lipid-rich samples. Together, these bioinformatics challenges associated with ATs omics necessitate specialized strategies for normalization, imputation, batch correction, and multi-omics integration to extract biologically meaningful insights. Furthermore, there is an ongoing difficulty in integrating multiple omic data sets (i.e., transcriptome, proteome, metabolome, and lipidome), and it is often difficult to establish the causal relationship between molecular changes and functional response. Limitations in sample accessibility, small-sized cohorts, and inter-individual variability limit the generalizability of human research findings. Therefore, to better understand of ATsmay be regulated in a systemic fashion through omics technology, future research should be multi-omic, longitudinal, and based upon relevant physiological models, thereby facilitating an understanding of the mechanisms that cause beige/brown-fat activation and the development of therapeutic strategies to address obesity and related disease.

## 6. Conclusions

ATs are far more than inert fat stores; they are dynamic, metabolically active, and highly communicative organs that orchestrate energy balance, thermogenesis, and systemic metabolism. Omics technologies have unveiled this hidden complexity, revealing the diverse cellular landscapes, depot-specific functions, and molecular choreography that drive white, brown, and beige fat biology. In WAT, multi-omics approaches have exposed intricate lipid remodeling, progenitor cell heterogeneity, and the molecular fingerprints of obesity and inflammation. In brown and beige fat, integrative omics have illuminated the tightly coordinated networks controlling thermogenesis, mitochondrial function, and adaptive energy metabolism, identifying novel regulators and potential therapeutic targets. Beyond deepening our mechanistic understanding, these discoveries underscore the systemic influence of ATs, linking molecular changes to whole-body metabolic health. While challenges remain, such as integrating multi-layered omics data, accounting for temporal dynamics, and translating animal findings to humans, the continued application of omics promises to transform our understanding of AT’s biology and provide innovative solutions for the treatment of obesity and other metabolic diseases.

## Figures and Tables

**Table 1:** Comparative Features of White, Brown, and Beige Adipose Tissue Types.

Feature	White Adipocyte	Brown Adipocyte	Beige Adipocyte
UCP1 expression	Negative	Positive	Positive (inducible)
Mitochondrial density	Low	High	Medium
Lipid droplet morphology	Unilocular	Multilocular	Multilocular (variable)
Primary function	Energy storage; endocrine activity	Thermogenesis; endocrine activity	Adaptive thermogenesis; endocrine-like activity
Cellular appearance	Single large lipid droplet, thin cytoplasm	Small droplets, mitochondria-rich cytoplasm	Intermediate between white and brown
Metabolic activity	Low	Very high	Moderate-high (stimulus-dependent)
Typical location	Subcutaneous and visceral fat depots	Dedicated depots	Within white adipose depots after stimulation
Plasticity	Stable phenotype	Stable phenotype	Highly plastic (can revert to white-like state)

## Data Availability

No new data were created or analyzed in this study.
